# Assessment of Public Special Education Teachers Training Needs on Evidence-Based Practice for Students with Autism Spectrum Disorders in Spain

**DOI:** 10.3390/children9010083

**Published:** 2022-01-06

**Authors:** Aitor Larraceleta, Luis Castejón, María-Teresa Iglesias-García, José Carlos Núñez

**Affiliations:** 1 Equipo Regional ACNEAE, Ministry of Education of the Principality of Asturias, Avenida San Pedro de los Arcos, 18, 33012 Asturias, Spain; 2 Department of Psychology, University of Oviedo, Plaza Feijoo s/n, 33003 Asturias, Spain; luiscf@uniovi.es (L.C.); jcarlosn@uniovi.es (J.C.N.); 3 Department of Educational Sciences, University of Oviedo, C/Aniceto Sela s/n, 33005 Asturias, Spain; teresai@uniovi.es

**Keywords:** autism, teachers education program, evidence-based practices, professional development

## Abstract

Over decades, the concern for the quality of psychoeducational practices for students with autism spectrum disorders has led to study to what extent are evidence-based educational methods disseminated among teachers. The purpose of this cross-sectional study, taking as reference Hsiao and Sorensen’s previous research, was to identify through a survey to what extent social-communication evidence-based practices for these students were provided in teacher education and in-service training programs, in a sample of 108 special education teachers from Spain, and to compare these results with Hsiao and Sorensen’s. Overall, more than 70% of the teachers reported that evidence-based practices in their teacher education programs (87.6%) and in-service training programs (73.6%) were *never taught or mentioned incidentally*. Finally, a higher percentage of addressing on each practice (i.e., *mentioned and discussed* or *mentioned and taught through direct instruction*) is shown in the sample of American teachers compared to the Spaniards, in both training paths.

## 1. Introduction

Over the last century, people with autism spectrum disorder (ASD), a lifelong neurodevelopmental disorder characterized by difficulties in social-communication skills and restricted and repetitive patterns of behaviors [1], have become increasingly present in different societies. For example, the growth in the percentage of people with ASD has gone from 0.05%, established in 1966, to the current range, between 0.9% and 1.5% of the population [2]. This reality translates into the educational field in the rising presence of students with ASD in schools [3] that need specialized educational accommodation due to their social and communicative particularities [4,5].

In the United States, between the school years 2011–2012 and 2018–2019, the number of students ages 3–21 who obtained special education services under the Individuals with Disabilities Education Act (IDEA) increased from 12.9% of total public-school registration (6.4 million) to 14.1% (7.1 million). During that period, students with ASD between those ages rose from 0.9% (455,000) to 1.5% (762,000), representing a 66.6% increase of students with ASD enrollment in public schools [6]. Cardinal et al. [7] point out that the rate of ASD growth has proceeded at a statically significant higher rate than have national changes in special education.

Given this reality, since the last decade of the last century, different international organizations and institutions have financed research through systematic reviews to identify the most effective models or practices in both educational and clinical intervention for people with ASD (f.e., [8,9,10,11]). Its main objective was to evaluate the efficacy of psychoeducational interventions concerning the improvement of the manifestations that define the autism spectrum [5]. Currently, in the educational field addressed to students with ASD, evidence-based practice (EBP) should consider child characteristics, intervention context, practitioner variables, and research-based knowledge [12]. Progressively, it has been incorporated into special education research [13,14,15,16], although their historical roots for students with ASD lie in the evidence-based medicine movement, which was born in England in the 1960s [5,11,17].

The educational intervention proposed to these students should be supported by teacher training aimed at reducing the gap between research on educational interventions and their implementation in the classroom [18], thus promoting the Implementation Science or transfer of practices that work in applied research to the school day [19]. In recent years, among the different investigations that have been undertaken, two comprehensive literature reviews have drawn the attention of the scientific literature: The National Autism Center (NAC) and The National Professional Development Center on ASD (NPDC) reviews [5,18]. In the mid-2000s, these two research teams conducted extensive and systematic reviews of the scientific literature on intervention in autism in parallel with a subsequent review five years later [20,21]. Both projects used systematic and rigorous criteria to evaluate the research: the methodological acceptability of each study located and had a set of highly qualified reviewers [21].

These teams confronted the studies identified in the others’ reviews, finding substantial consensus between them [22]. It is important to highlight, and hence the choice of these two proposals as a model for our study, that although at that time there were other systematic reviews of individual practices (f.e., [23]), none of them carried out exhaustive reviews of the diversity of behavioral and developmental intervention practices in the scientific publications for children and youth with ASD in the period ranging from infancy to 22 years old [21].

The NAC’s National Standards Project [9] is an unprecedented multi-year project, establishing a body of standards for research-validated and effective educational and behavioral interventions for children on the spectrum, with the support and advice of an expert panel comprised of academics, researchers, and other recognized leaders in the U.S. representing diverse fields of study. These standards identify psychoeducational interventions focused effectively on the core symptoms of ASD.

In 2015, the NAC presented a new review and analysis based on research conducted in the domain between 2007 and 2012 [24]. Fourteen established evidence-based practices were selected, although some of them are sets of practices: “behavioral interventions”, “cognitive-behavioral intervention package”, “comprehensive behavioral treatment for young children”, “language training (production)”, “modeling”, “natural teaching strategies”, “parent training”, “peer training package”, “pivotal response training”, “schedules”, “scripting”, “self-management”, “social skills package”, and “story-based intervention” [24].

The proposal in charge of the National Professional Development Center on Autism Spectrum Disorders was a program financed by the Office of Special Education Programs of the United States Department of Education and developed in collaboration with three universities: the University of Carolina del Norte in Chapel Hill, the University of Wisconsin in Madison, and the MIND Institute of the University of California at Davis [11]. Its objective is to promote the use of evidence-based strategies for children and young people with autism, from birth to 22 years of age, through a comprehensive professional development process of the professionals who work with them in fields such as education, intervention, early detection, or diagnosis [25].

Therefore, teachers and other professionals such as psychologists, speech therapists, etc., select these practices when designing an educational program or individualized intervention because scientific evidence endorses its contribution to the achievement of results similar to the developmental goals established for students without autism [26]. For this purpose, the NPDC on ASD has designed a process through which these strategies could be used systematically in early intervention programs and at school [27], through online training modules for teachers, such as the Autism Focused-Intervention Resources and Modules [28] or the Autism Internet Modules [29].

In 2014, this project carried out a broad initial search that produced a corpus of 29,106 papers associated with ASD and its intervention, framed between 1990 and 2011 [5,11,18]. Of these articles, 456 met the inclusion criteria and, from them, a list of strategies and focused behavioral interventions was extracted, as well as another diverse group of techniques and interventions that could be useful in the educational and therapeutic fields [30], giving a final result of 27 focused intervention practices that satisfy evidence-based criteria [11], and which include, for example, “antecedent-based interventions”, “discrete trial training”, “modeling”, “naturalistic intervention”, “peer-mediated instruction and intervention”, “prompting”, “reinforcement”, “social narratives”, or “video modeling”. These represent three more than those identified in a previous review in 2007 [31].

### 1.1. Evidence-Based Practices for Students with ASD

To respond to the social challenge posed by ASD in Spain, in 2015, the Ministry of Health, Social Services, and Equality approved the Spanish Strategy on Autism Spectrum Disorders [32], a reference framework in the definition of state, regional, and local policies and actions aimed at people with ASD. This framework establishes 15 specific strategic lines, dedicating the ninth of them to education. Among the eleven objectives that this strategic line raises, it seems important to highlight two, due to their relationship with the theme of this research [33] (pp. 50–51): “Objective 1: Promote knowledge about the situation and needs of students with ASD in Spain in the different educational stages, developing or reactivating the initiatives that are necessary for this and promoting the necessary actions (good practices, evidence-based practices, dissemination of recommended methodologies, etc.)” and “Objective 6: Promote educational innovation networks, as well as the development, evaluation, and implementation of good practices based on evidence, as well as the dissemination of teaching methodologies and systems that have demonstrated greater effectiveness in targeted educational intervention to students with ASD”.

Recently, a general collaboration protocol has been signed between the Ministry of Education of Spain and Autism Spain to improve educational care in search of ensuring a successful transition to adult life for the almost 50,000 schoolchildren with ASD and enhancing their quality of life [34,35]. These children grew in number by 160% between the school years 2011–2012 and 2018–2019 [35], an almost identical percentage to American schoolchildren [8], and show an estimated prevalence of “1.55% in preschoolers and 1% in school-age children” in Spain [36]. Among the actions contemplated is the promotion of “networks aimed at the development and implementation of good practices based on evidence and educational innovation” [34], highlighting once again the importance of incorporating practices based on the evidence within the educational response to these students.

For the proper functioning of these networks, the role played by special education teachers will be crucial. In Spain, these teachers are divided into two specialties, Hearing and Language (known in Spanish as “Audición y Lenguaje-AL”) and Therapeutic Pedagogy (known in Spanish as “Pedagogía Terapéutica-PT”) and work mainly with students considered to have special educational needs (SEN) [37], including students with ASD. Their work, for example, can be aimed at advising general teachers who, for the most part, do not feel prepared to respond to pupils with ASD [38,39].

The educational intervention in both specialties is related to social communication, which is defined as:

“A child’s understanding of speaker intentions and the verbal and nonverbal cues that signal those intentions, as well as the child’s interpretation of the environmental context, societal norms, and expectations and how these coalesce with structural aspects of language (e.g., vocabulary, syntax, and phonology) to achieve successful communication” [40].

Therefore, the justification for this study about social-communication evidence-based practices for the students with ASD would be fourfold:

− The right of all people to education—which is included in article 27 of the Spanish Constitution- and how this education should be directed to achieve its full potential [32].
− The signs of lack of specific teacher training on ASD in Spain [30,39].
− The risks associated with the lack of successful results for students with ASD and their families and teachers (poor educational results, teacher burnout, family concern...) [18,41].
− The historical tradition of interventions without a scientific basis has been publicly available in the educational field [18].

### 1.2. Purpose of the Study

The purpose of this study was to identify to what extent social-communication evidence-based practices for students with ASD were provided in teacher education and in-service training programs in a sample of special education teachers of pupils with ASD in the north of Spain following, as a reference, Hsiao and Sorensen Petersen’s previous research [13] to compare the instructional situation in the United States versus that in Spain. Thus, it helps to respond to one of the limitations of the original study: a greater variety of demographic variables from a cross-cultural approach. The next research questions were addressed:

Research Question 1: to what extent was the training in social-communication evidence-based practice provided in teacher education programs for north Spanish special education teachers of students with ASD?

Research Question 2: to what extent was the in-service professional development on social-communication evidence-based practice provided to north Spanish special education teachers of students with ASD?

Research Question 3: What is the training status of Spanish teachers’ sample compared to the teachers in the US research?

## 2. Materials and Methods

### 2.1. Study Design and Participants

An observational cross-sectional study was employed to answer the research questions. The population was recruited in Asturias, a region located in the north of Spain with 1,022,800 people in 2019 [42], where 7% of the school population with special education needs (SEN) is associated with autism spectrum disorder [43], and there has been an increase of 513% of these pupils over the past decade (the academic year 2007–2008 to 2017–2018) [44].

The population for this study was collected, on the one hand, from the eight special education schools, that enroll students with ASD and, on the other hand, from special education teachers in ordinary schools who participated in training actions of centers of teachers and resources, agencies responsible for teacher professional development. A total of 108 special education teachers in public schools responded to this research (the responses from nine private school teachers and eight teachers who did not work as special education teachers were discarded). This sample represents close to 15% of the total number of special education public teaching staff in the region who totaled 726 two years ago [45].

All participants’ characteristics are shown in Table 1. Among these 108 teachers, most participants were female (89.8%), between the ages of 36–50 years old, and had achieved a university degree—Bachelor qualification or equivalent according to the European Qualification Framework−(89.8%). Of those surveyed, the most frequently chosen option was “had been working as a teacher between 11–20 years” (35.2%), “had been teaching students with ASD between 1–5 years” (43.5%), and “between 1–5 children with ASD” (32.4%) and, lastly, “had been receiving specific training about ASD provided in in-service professional development and other sources and absence in teacher education programs” (42.6%).

### 2.2. Instrument

A survey study was carried out using a questionnaire (based on the previous research by Hsiao and Sorensen-Petersen [13] and the publications of the NPDC on ASD [11] and the NAC [9]. For its development, those focused practices that coincided in the NAC and NPDC reviews were selected [11,24], obtaining 21 results and leaving out six: “exercise”, “functional behavior assessment”, “functional communication training”, “picture exchange communication system”, and “structured playgroups” [22].

Subsequently, an inclusion criterion for the remaining 21 practices was established consisting of the evidence of their effect at some point between birth and 21 years, in three areas of evolutive development, related to the social-communicative dimension of ASD: social, communicative, and joint attention [5,24]. Twelve results were obtained and included in the items of the questionnaire: “differential reinforcement”, “discrete trial teaching”, “modeling”, “naturalistic intervention”, “peer-mediated intervention”, “prompting”, “reinforcement”, “scripting”, “social narratives”, “task analysis”, “time delay”, and “video modeling”. For all participants to have a shared understanding of each strategy, the short definitions from the NPDC were included. Teachers rated each item about the training provided in teacher education programs or in-service training/professional development using a 4-point Likert-type scale (*never mentioned and never taught* = 1, *mentioned incidentally* = 2, *mentioned and discussed* = 3, and *mentioned and taught through direct instruction* = 4), replicating Hsiao and Sorensen Petersen’s proposal [13]. There was one question dedicated to the general assessment of the training on the set of evidence-based practices selected in this study received in teacher education programs or in-service training/professional development using a different 4-point Likert-type scale (*very unsuitable* = 1, *unsuitable* = 2, *suitable* = 3, and *very suitable* = 4). Other survey items regarding teacher demographics were included in the questionnaire and reflected in Table 1. The final instrument is presented at the end of this work in both English (Appendix A) and Spanish (Appendix B). Finally, the questionnaire was converted to an online format using Microsoft Forms^©^ for its elaboration and dissemination. It explicitly warned that its completion implied consent to participation. The internal consistency of the questionnaire (*k* = 26; *N* = 108), estimated using Cronbach’s alpha coefficient [46] was 0.907, rated as excellent [47].

### 2.3. Procedures

The study procedures were evaluated and approved by the Ethics Committee of the University of Oviedo (vRTIual_learning70/18). A non-probabilistic sampling technique was carried out in a snowball format [48] to obtain the highest participation of special education teachers who currently work or have worked with students with ASD. Online surveys were sent to the principals of the eight special schools that educate students with ASD in the region and were disseminated among their teachers. Online surveys were also sent to the Diversity Advisers of the four Centers of Teachers and Resources of the Asturian Ministry of Education, who disseminated them among special education teachers who participated in their training sessions. The survey completion period was one month.

### 2.4. Analysis

As in the study by Hsiao and Sorensen-Petersen [13], the number and percentage of responses were calculated to pose how the training was provided to the teachers on each and in the set of evidence-based practices, in teacher education programs and in-service professional development. We also use descriptive statistics (i.e., means, standard deviations) to summarize the teachers’ ratings about the training provided on each evidence-based practice selected in this research and to the set of practices.

## 3. Results

### 3.1. Training on Evidence-Based Practices in Teacher Education Programs

The number and percentage of responses about training on the twelve evidence-based practices selected in this research are presented in Table 2. Regarding each evidence-based practice in teacher education programs, the participants indicated, as the most selected option (in eight on the twelve practices with percentages at least close to 50%), that the educational strategies proposed *were never mentioned* and *never taught*: video modeling (86.1%), discrete trial training (70.4%), time delay (65.7%), naturalistic intervention (59.3%), peer-mediated interventions (59.3%), scripting (56.5%), social narratives (52.8%) and differential reinforcement (49.1%). In the case of the four remaining practices, the most selected option was mentioned incidentally: prompting (57.4%), modeling (53.7%), task analysis (49.1%), and reinforcement (47.2%). On the contrary, in eleven of the twelve practices—except reinforcement (3.7%), either no participant received direct instruction in their teacher education program—differential reinforcement, discrete trial training, modeling, peer-mediated interventions, prompting, time delay, and video modeling—or only one teacher (0.9%) received it—naturalistic intervention, scripting, social narratives, and task analysis.

According to our research, 47.54% of the participants reported that the set of practices *never was taught* and 40.07% reported that they *were mentioned incidentally*; 11.70% *were mentioned and discussed*, and only 0.59% said that these practices *were taught through direct instruction*. Overall, only 12.29% reported that the strategies were either *taught through direct instruction or discussed* in their teacher education programs.

Additionally, if the percentages in each evidence-based practice are considered, more than 80% of participant teachers reported—with the exceptions of modeling (63.9%) and reinforcement (51.8%)—that they *were never taught or just mentioned incidentally*: video modeling (86.1% + 13% = 99.1%), time delay (65.7% + 33.3% = 99%), discrete trial training (70.4% + 26.9% = 97.3%), peer mediated interventions (59.3% + 37% = 96.3%), naturalistic intervention (59.3% + 36.1% = 95.4%), social narratives (52.8% + 41.7% = 94.5%), differential reinforcement (49.1% + 45.4% + 94.5%), scripting (56.5% + 36.1% = 92.6%), task analysis (39.8% + 49.1% = 88.9%) and prompting (26.9% + 57.4% = 84.3%).

Overall, regarding mean scores obtained in each evidence-based practice in teacher education programs, teachers expressed low levels of training received in ten of the twelve strategies –individual item means ranged from 1.15 to 2.47; Table 3: prompting (*M* = 1.89), task analysis (*M* = 1.72), differential reinforcement (*M* = 1.56), social narratives (*M* = 1.54), scripting (*M* = 1.52), naturalistic intervention (*M* = 1.46), peer mediated interventions (*M* = 1.44), time delay (*M* = 1.35), discrete trial training (*M* = 1.32), and video modeling (*M* = 1.15). Participants only reported moderate levels of perceived training in two of the evidence-based practices evaluated in this study: reinforcement (*M* = 2.47) and modeling (*M* = 2.26).

The overall rating about the evidence-based practices proposed in this research in teacher education programs expressed low levels of satisfaction (*M* = 1.45, *SD* = 0.519) about their training: 55.6% of the participating teachers rated training at this stage as *very unsuitable*; 43.5% as *unsuitable* and only 1% as *suitable* (Table 4).

### 3.2. Training on Evidence-Based Practices in In-Service Professional Development

The number and percentage of responses about training on the twelve evidence-based practices selected in this research are presented in Table 5.

Regarding each evidence-based practice in in-service professional development, the participants reported as the most selected option (in ten on the twelve practices with percentages, all of them greater than 40%), that the educational strategies proposed *were mentioned incidentally*: differential reinforcement (63%), prompting (57.4%), task analysis (56.5%), scripting (53.7%), discrete trial training (53.7%), social narratives (50.9%), peer-mediated interventions (50.9%), naturalistic intervention (47.2%), reinforcement (43.5%) and modeling (41.7%). In the case of the two remaining practices –video modeling (66.7%) and time delay 42.6%—the most selected option was *never mentioned and never taught*.

Conversely, in each of the twelve practices, less than 8% of the participant teachers reported that the strategy *was mentioned and taught through direct instruction*: video modeling and discrete trial training (1.9%), differential reinforcement (2.8%), peer-mediated interventions, prompting and time delay (4.6%), naturalistic intervention, reinforcement, social narratives, and task analysis (5.6%), scripting (6.5%), and modeling (7.4%).

According to our research, while 48.15% of participants reported that the practices *were mentioned incidentally* and 25.51% of teachers reported that the set of practices *was never taught*, 21.80% reported that they *were mentioned and discussed* and only 4.55% reported that these practices *were taught through direct instruction*. Overall, only 26.35% indicated that the strategies were either *taught through direct instruction or discussed* in their teacher education programs.

Additionally, if we consider the percentages in each evidence-based practice, more than 60% of participants teachers reported—with the exceptions of modeling (51%) and reinforcement (51.8%)—that they *were never taught or just mentioned incidentally*: video modeling (66.7% + 25% = 91.7%), discrete trial training (33.3% + 53.7% = 87.0%), differential reinforcement (20.4% + 63% + 83.4%), time delay (42.6% + 39.8% = 82.4%), peer mediated interventions (27.8% + 50.9% = 78.7%), prompting (17.6% + 57.4% = 75%), naturalistic intervention (26.9% + 47.2% = 74.1%), task analysis (13.9% + 56.6% = 70.4%), scripting (15.7 + 53.7 = 69.4%), and social narratives (13.9% + 50.9% = 64.8%).

Overall, regarding mean scores obtained in each evidence-based practice in in-service professional development, teachers expressed low levels of training received only in five of the twelve strategies—individual item means ranged from 1.44 to 2.47; Table 3: differential reinforcement (*M* = 1.99), peer mediated interventions (*M* = 1.98), discrete trial training (*M* = 1.81), time delay (*M* = 1.80) and video modeling (*M* = 1.44). Participants report moderate levels of training received in the rest of the evidence-based practices evaluated in this study: modeling (*M* = 2.47), reinforcement (*M* = 2.45), social narratives (*M* = 2.27), task analysis (*M* = 2.21), scripting (*M* = 2.21), prompting (*M* = 2.12), and naturalistic intervention (*M* = 2.05).

The overall rating about the evidence-based practices proposed in this research in in-service professional development expressed moderate levels of satisfaction (*M* = 2.33, SD = 0.697) about their training: 8.3% of the participating teachers rated training at this stage as *very unsuitable*; 54.6% as *unsuitable*; 32.4% as *suitable* and 4.6% as *very suitable* (Table 4).

### 3.3. Comparison between the Training Status of the Spanish Sample Compared to the US Research

It is necessary to answer the third research question, to compare the results of the research by Hsiao & Sorensen Petersen [13] with the results of our study to approach the training status of Spanish special education teachers.

Regarding training on evidence-based practices in teacher education programs, the eleven practices that coincide between both studies (scripting is not included in the US research), in an overwhelmingly higher percentage, have been taught through direct instruction or discussed in the sample of teachers in the study by Hsiao and Sorensen-Petersen [13] than in our study. Thus, prompting obtained 87.3% in the US teacher sample versus 17.7% in the Spanish sample; task analysis obtained 81% versus 11.1%; discrete trial training obtained 63.5% versus 2.8%, naturalistic intervention obtained 62.3% versus 4.6%; differential reinforcement obtained 61.4% versus 5.6%, social narratives obtained 55.6% versus 5.5%; reinforcement obtained 93.7% versus 48.1%; modeling obtained 81% versus 36.1%, time delay obtained 44.5% versus 0.9%; peer-mediated interventions obtained 39.7% versus 3.7% and finally, video modeling obtained 31.7% versus 0%.

If total results concerning evidence-based practices are compared, the percentage that has been *taught through direct instruction* or *discussed* in the sample of teachers in the study by Hsiao and Sorensen-Petersen [13] amounts to 63.7%, versus 12.3% in our study.

Regarding training on evidence-based practices in in-service professional development, the eleven practices that match in both pieces of research, in a higher percentage, have been *taught through direct instruction or discussed* in the sample of American teachers than in our study. Thus, prompting obtained 81.0% in the US teacher sample versus 25.0% in the Spanish sample; discrete trial training obtained 65.1% versus 13%; differential reinforcement obtained 63% versus 16.7%; task analysis obtained 74.6% versus 29.7%; video modeling obtained 44.5% versus 8.4%, reinforcement obtained 84.1% versus 48.2%; social narratives obtained 69.8% versus 35.2%; naturalistic intervention obtained 54% versus 26%; time delay obtained 39.7% versus 17.6%; modeling obtained 68.3% versus 49.1% and finally, peer-mediated interventions obtained 39.7% versus 17.2%.

With regard to compared total results about evidence-based practices, the percentage that has been *taught through direct instruction or discussed* in the sample of teachers in the study by Hsiao and Sorensen-Petersen [13] amounts to 61.6% versus 26.4% of Spanish teachers.

## 4. Discussion

The purpose of this study was to identify to what extent social-communication evidence-based practices for students with ASD were provided in teacher education and in-service training programs in a sample of special education teachers of pupils with ASD in Spain.

Regarding the first research question, 87.6% of the participants informed that the selected evidence-based practices in their teacher education programs either *were never taught* (47.5%) or *were mentioned incidentally* (40.1%) (Table 2). These results indicate percentages much higher than those obtained by Hsiao and Sorensen-Petersen [13], in which 36.3% (17.4% + 18.8%) of the teachers of their study had not received training in this training pathway or only incidentally. Furthermore, the sample of Spanish teaching staff values 99.1% of the practices in their teacher education programs like *unsuitable* or *very unsuitable* (Table 4). The results indicate the need to review this training path. 

These results on the evidence-based practices evaluated in this study seem to go further and be related to the overall lack of training on ASD in the university-based teacher training programs and are aligned with the research of Morrier et al. [49], which established that less than 15% of the teachers received training from university-based teacher preparation programs. Specifically, 66.7% of Spanish special education teachers who participated in our research (Table 1) reported that they had not received any training on ASD in teacher education programs, and more specifically, 14.8% had not received any training on ASD in any training path. These results would be in line with those of Saldaña et al. [50] who, in one of the few studies carried out in Spain on evidence-based practices, concluded that an effort was necessary for Andalusian schools (south of Spain) to expand the specific knowledge base that teachers had on the effectiveness of the interventions available for students with autism. Regarding the analysis of each of the evidence-based practices selected in this study concerning the teacher education programs (Table 3), it should be noted that, although the score for each strategy is low, two practices seem to have been taught with more dedication during these programs: reinforcement (*M* = 2.47, *SD* = 0.632) and modeling (*M* = 2.26, *SD* = 0.632). One of the possible reasons for this difference could be that these conventional behavioral practices [30] are more deeply rooted in Spanish university-based teacher preparation programs than those, for example, that use technology (e.g., video modeling). This lack of teacher training on practices that involve the use of technology is also observed in the study by Hsiao and Sorensen [13], although not so markedly.

Regarding the second research question, 73.6% of the participants reported that the selected evidence-based practices in their in-service professional development either *never were taught* (25.5%) or *were mentioned incidentally* (48.1%) (Table 5). These results also indicate percentages much higher than those obtained by Hsiao and Sorensen-Petersen (2019). In this research, 37.9% (20.4% + 17.4%) of the teachers of their study had not received training in their training pathway or only incidentally. Besides, 62.9% of the sample of Spanish teaching staff values their in-service professional development as *unsuitable* or *very unsuitable* (Table 4). The results point out that the review of these training avenues is necessary, thus responding to the demands of the teachers to be able to offer an adequate educational response to their students with ASD since the possibility of finding quality in-service training on EBPs in ASD is scarce [19]. However, 81.5% of Spanish teachers reported training on ASD at some point in their in-service professional development, either exclusively (7.4%) or in combination with other training pathways (Table 1). Overall, this training avenue shows better results than teacher education programs, although not enough to overcome the challenges posed by the education of these students.

About the analysis of each of the evidence-based practices selected in this study concerning in-service professionals (Table 3), although the scores, in general, are a little higher than in the previous training pathway, the pattern is repeated: modeling (*M* = 2.47, *SD* = 0.767) and reinforcement (*M* = 2.45, *SD* = 0.728) obtain the highest scores, which seems to underpin the explanation provided in the previous section.

Regarding the last research question, the comparison between the results of both studies shows a higher level of training on evidence-based practice (*taught through direct instruction or discussed*) in the sample of American teachers in all the practices that coincide between the two studies. The difference in teacher education programs ranges from 31.7% to 71.6%: prompting (71.6%), task analysis (69.9%); discrete trial training (60.7%), naturalistic intervention (57.7%); differential reinforcement (55.8%), social narratives (50.1%); reinforcement (45.6%); modeling (44.9%), time delay (43.6%); peer-mediated interventions (36%) and video modeling (31.7%).

In in-service professional development, differences ranged from 18.4% to 56%: prompting (56%), discrete trial training (52.1%), differential reinforcement (46.3%), task analysis (44.9%), video modeling (36.1%), reinforcement (35.9%), social narratives (34.6%), naturalistic intervention (28%), time delay (22%), modeling (19.2%) and peer mediated interventions (18.4%).

When looking at the total results, the percentage difference between those practices that *were taught through direct instruction* or *discussed in teacher education programs*, in the sample of American teachers in the Hsiao and Sorensen Petersen research [13] and our study exceeds 50% (51.4%).

If we look at the total results in in-service professional development, the percentage difference between those practices that *were taught through direct instruction or discussed* in the sample of American teachers and our study is somewhat lower (35.2%) but still too high.

The set of previous conclusions highlights a recurring fact in the scientific literature that must be transferred to university-based teacher preparation programs and teacher professional development, especially in Spain: the increase in the presence of students with ASD in classrooms must be accompanied by specific and specialized training of special education teachers based on high-quality research, [17,18,21,51,52,53,54]. This need becomes peremptory, especially:

− When scientific evidence indicates that the majority of teachers express their desire to provide evidence-based interventions and one of their principal concerns is the possible lack of training and preparation in this area by university programs [5,18,20,55].
− When such training and the sustained use of evidence-based strategies are a priority, as fidelity in the use of these practices is related to better results [18,51,54].
− When research indicates that a significant part of the special education teachers continues to use practices with a lack of evidence or with no such evidence [13,52,55], trusting, like other professionals, only in the so-called “practice wisdom” [55].
− When the scientific literature reports that teacher burnout stands as an issue in special education, and also indicates that the implementation of a high-quality program that employs EBP may be related to less burnout. The use of strategies based on the evidence shows higher levels of teacher self-efficacy, which dampens said burnout [21].
− And finally, when there is a growing demand for a more equitable proportion of appropriate educational services to students with ASD by families [53,56].

There are some limitations to this pilot study that should be taken into consideration.

First, we recognize the sample selection process itself as a limitation, which is why more studies with other independent samples would be needed to confirm our results. Since in our study we have a convenience sample, an independent one would improve the quality of the study, but this is logistically difficult due to the problems of determining the exact number of teachers during the academic year and contacting them, especially across the COVID pandemic. In any case, our sample seems large enough to consider the results obtained, although the study replication would be of interest.

Second, in the present study, the data have been treated without considering the potential influence of the gender variable. It has been the case since the sample was unbalanced (i.e., 90% of the participants are female). Analyzing the effect of gender in this way could include more noise than certainties. However, future studies should consider analyzing the effect of this variable, for example, on the use of different teaching standards.

Third, in the sample of this study, there are hardly any young teachers (e.g., with less than one year of experience or under 25 years old which has not allowed us to know the effect of the degree of experience on the choice of teaching strategies. It is possible that professionals with 20 or more years of practice were educated during a period when specific autism strategies weren’t probably included in the university curriculum in many universities worldwide. However, it is also possible that there are no significant differences in the professional practices of young and older teachers [49,57], perhaps because of the continuous training received. In any case, this seems a matter of real importance that deserves to be rigorously investigated in future studies.

Fourth, it is possible that despite providing definitions of each evidence-based practice, some participants might have misunderstood some of the strategies selected, for example, confusing social narratives, a set of practices that include, among others, social stories, power cards, and cartooning, with social stories, a concrete social narrative.

Fifth, in this research, the survey methodology is used, and therefore the information offered by the teachers about their training in evidence-based practices is collected which may or may not describe the real implementation of EBP strategies, since self-report is many times related as a source of possible bias. Other observational data would be helpful to specify whether the self-report data were reflected in the classroom and supplied information about the fidelity with each practice that was being implemented.

Finally, it would be interesting if the set of evidence-based practices used for this study was extended to all evidence-based practices and the new selected practices obtained in the NPDC research (currently National Clearinghouse on Autism Evidence and Practice) [13] and in the NAC’s new National Standards Report, expected in 2021.

Despite their limitations, the findings of this research may have different implications for educational practices and policies.

First, about 90% (Table 2) of respondents have received little or no training on the evidence-based practices evaluated in this study during their teacher education programs and more than 99% of surveyed rated the overall training on evidence-based practices received in these programs as *unsuitable* or *very unsuitable* (Table 4).

Second, about 75% of those surveyed (Table 5) have received little or no training in their in-service professional development on the evidence-based practices evaluated, and 63% of those surveyed rated the overall training received on these practices as *unsuitable* or *very unsuitable* (Table 4). Furthermore, about 70% of the teachers (Table 1) had not received any training on ASD in university programs, and almost 15% (Table 1) by no training path.

Third, the teachers surveyed rated the training they have received better in their in-service professional development than in their teacher education programs (Table 3). If the differences between each of the twelve practices in the two paths are observed, the training received in eleven of the twelve obtain a better value in professional development than in the university-based teacher preparation programs (except reinforcement that achieves a score practically the same in both paths). However, according to the results, in none of the cases have they been discussed in-depth or by direct instruction.

These three findings imply that there is still a long way to go in incorporating these practices in university training programs and professional development. It cannot be forgotten that this situation occurs in a context in which the presence of these students in Spanish classrooms has grown exponentially and the need to provide an adequate educational response through better preparation of special education teachers who work with learners with ASD is “imperative” [18] (p. 10), [51] (p. 29) and a common conclusion in many previous studies. The knowledge, training, selection, and implementation of evidence-based practices play a crucial role in it [51].

Therefore, our results indicate the imperative need for the review of university training and professional development programs and the incorporation of evidence-based practices to support Spanish special education teachers in their knowledge and methodology development focused on teaching students with ASD.

## 5. Conclusions

We concluded that in the context of the study, teacher training should have as its purpose something that for the teachers is insufficient, which is the dissemination of knowledge about EBP. Teachers have predominantly characterized their initial and in-service training as “unsuitable” or “very unsuitable”. If this is the case, from this study, we can conclude that it would be necessary to rethink the current training on the education of the students with ASD to ensure that the teachers know the EBP. Thus, it would be possible to develop an effective and efficient educational intervention. For this purpose, it is also relevant to deepen the relationship between the quality of the actions of the teaching staff and the quality of the training they receive, helping to bridge the educational research and practice gap. On the other hand, the comparison of the results obtained with previous studies carried out in the USA raises the idea that, in our context, there is an area of improvement in the performance of special education teachers. This improvement would be possible to accomplish, disseminating in teacher education programs and in-service training the knowledge of EBP for students with ASD.

## Figures and Tables

**Table 1:** Participant Demographics.

Characteristics	Number of Teachers (%)
Gender	
Female	97 (89.8)
Male	11 (10.2)
Age bracket (years)	
Under 25	3 (2.8)
26–35	25 (23.1)
36–50	65 (60.2)
Over 50	15 (13.9)
Highest academic qualification (European Qualification Framework)	
Bachelor or Equivalent	97 (89.8)
Master	11 (10.2)
Number of years in the profession	
Less than one year	4 (3.7)
1–5	24 (22.2)
6–10	25 (23.1)
11–20	38 (35.2)
21 or more	17 (15.7)
Total number of years teaching students with ASD	
Less than one year	7 (6.5)
1–5	47 (43.5)
6–10	35 (32.4)
11–20	15 (13.9)
21 or more	4 (3.7)
Total number of students with ASD	
1–5	35 (32.4)
6–10	28 (25.9)
11–20	30 (27.8)
21 or more	15 (13.9)
Sources of specific training about ASD	
No specific training	16 (14.8)
Specific training provided in In-Service Professional Development and other sources (e.g., ASD organizations) and absence in Teacher Education Programs	46 (42.6)
Specific training provided in Teacher Education Programs and other sources and absence in In-Service Professional Development	2 (1.9)
Exclusively in In-Service Professional Development	8 (7.4)
Exclusively in other sources (e.g., ASD organizations) or self-training	2 (1.9)
Miscellany: specific training provided in teacher education Programs and In-Service Professional Development with the presence of other sources.	34 (31.5)

**Table 2:** Type of Training of Identified Evidence-Based Practices Provides in Teacher Education Programs.

	Number (Percentage) of Responses			
	The Strategy			
Evidence-Based Practices	Was Never Mentioned and Never Taught	Was Mentioned Incidentally	Was Mentioned and Discussed	Was Mentioned and Taught through Direct Instruction
Differential reinforcement	53 (49.10)	49 (45.40)	6 (5.60)	-^1^
Discrete trial training	76 (70.40)	29 (26.90)	3 (2.80)	-
Modeling	11 (10.20)	58 (53.70)	39 (36.10)	-
Naturalistic intervention	64 (59.30)	39 (36.10)	4 (3.70)	1 (0.90)
Peer-mediated interventions	64 (59.30)	40 (37.00)	4 (3.70)	-
Prompting	29 (26.90)	62 (57.40)	17 (15.70)	-
Reinforcement	5 (4.60)	51 (47.20)	48 (44.40)	4 (3.70)
Scripting	61 (56.50)	39 (36.10)	7 (6.50)	1 (0.90)
Social narratives	57 (52.80)	45 (41.70)	5 (4.60)	1 (0.90)
Task analysis	43 (39.80)	53 (49.10)	11 (10.20)	1 (0.90)
Time delay	71 (65.70)	36 (33.30)	1 (0.90)	-
Video modeling	93 (86.10)	14 (13.00)	1 (0.90)	-
Total	566 (47.54)	476 (40.07)	139 (11.70)	7 (0.59)

^1^ The symbol “-” represents “no answers”.

**Table 3:** Mean Scores (*N* = 108) in Teacher Education Programs and In-Service Professional Development.

Evidence-Based Practices	*M*	*SD*	*M*	*SD*
Differential reinforcement	1.56	0.600	1.99	0.677
Discrete trial training	1.32	0.526	1.81	0.699
Modeling	2.26	0.632	2.47	0.767
Naturalistic intervention	1.46	0.618	2.05	0.836
Peer-mediated interventions	1.44	0.569	1.98	0.797
Prompting	1.89	0.646	2.12	0.745
Reinforcement	2.47	0.648	2.45	0.728
Scripting	1.52	0.662	2.21	0.786
Social narratives	1.54	0.633	2.27	0.769
Task analysis	1.72	0.681	2.21	0.749
Time delay	1.35	0.499	1.80	0.840
Video modeling	1.15	0.382	1.44	0.701

**Table 4:** Overall Rating (*N* = 108) about Evidence-Based Social-Communication Focused Practice in Teacher Education Programs and In-Service Professional Development.

	Overall Rating		Number (Percentage) of Responses			
	*M*	*SD*	Very Unsuitable	Unsuitable	Suitable	Very Suitable
In Teacher Education Programs	1.45	0.519	60 (55.60)	53 (43.50)	1 (0.90)	-^1^
In In-Service Professional Development	2.33	0.697	9 (8.30)	59 (54.60)	35 (32.40)	5 (4.60)

^1^ The symbol “-” represents “no answers”.

**Table 5:** Type of Training of Identified Evidence-Based Practices Provides in In-Service Professional Development.

	Number (Percentage) of Responses			
	The Strategy			
Evidence-Based Practices	Was Never Mentioned and Never Taught	Was Mentioned Incidentally	Was Mentioned and Discussed	Was Mentioned and Taught through Direct Instruction
Differential reinforcement	22 (20.40)	68 (63.00)	15 (13.90)	3 (2.80)
Discrete trial training	36 (33.30)	58 (53.70)	12 (11.10)	2 (1.90)
Modeling	10 (9.30)	45 (41.70)	45 (41.70)	8 (7.40)
Naturalistic intervention	29 (26.90)	51 (47.20)	22 (20.40)	6 (5.60)
Peer mediated interventions	30 (27.80)	55 (50.90)	18 (16.70)	5 (4.60)
Prompting	19 (17.60)	62 (57.40)	22 (20.40)	5 (4.60)
Reinforcement	9 (8.30)	47 (43.50)	46 (42.60)	6 (5.60)
Scripting	17 (15.70)	58 (53.70)	26 (24.10)	7 (6.50)
Social narratives	15 (13.90)	55 (50.90)	32 (29.60)	6 (5.60)
Task analysis	15 (13.90)	61 (56.50)	26 (24.10)	6 (5.60)
Time delay	46 (42.60)	43 (39.80)	14 (13.00)	5 (4.60)
Video modeling	72 (66.70)	27 (25.00)	7 (6.50)	2 (1.90)
Total	303 (25.51)	572 (48.15)	259 (21.80)	54 (4.55)

## Data Availability

The data presented in this study are available on request from the corresponding author. The data are not publicly available due to their containing information that could compromise the privacy of research participants.

## Appendix A

Sociodemographic Data

What is your gender? *Male *Female
*What is your current age? Under 25 (25 y. o. or less) Between 26–35 (35 y. o. included) Between 36–50 (50 y. o. included) Over 50 (51 y. o. and older)
*What is the highest academic degree you have achieved? Bachelor or Equivalent (Diplomatura Universitaria) Bachelor or Equivalent (Licenciatura Universitaria) Bachelor or Equivalent (Grado Universitario)
Master Doctorate Other
*What type are the ownership of the school where you work and your employment situation? Public: career official Public: probationary official Public: interim official Concerted: hired Private: hired
*What is the teaching specialty in which you are currently active? Hearing and Language (Special Education) Therapeutic Pedagogy (Special Education) Other specialties:
*Including this school year, what is your work experience in the teaching field? Less than 1 year Between 1 and 5 years (both included) Between 6 and 10 years (both included) Between 11 and 20 years (both included) 21 years or older
*Including this school year, how many years have you worked with students with ASD throughout your professional career? Less than 1 year Between 1 and 5 years (both included) Between 6 and 10 years (both included) Between 11 and 20 years (both included) 21 years or older
*Including this school year, how many children with ASD have you worked with throughout your professional career? None Between 1–5 Between 6–10 Between 11–20 21 or more
*Have you received any specific training on ASD? Yes No
*Where have you received this specific training on ASD (multiple responses)? In my university career In university extension courses or workshops In teacher training courses or workshops In courses or workshops taught by other organizations Through self-training: books, articles, etc. Others

Evidence-Based Practice Data

*How would you globally assess the training you have received in your university stage on evidence-based practice to respond to the Special Educational Needs in Social Communication of students with ASD? 1. Very Unsuitable 2. Unsuitable 3. Suitable 4. Very Suitable
*How would you globally assess the training you have received within the teacher training programs (CPR etc.) on evidence-based practice to respond to the Special Educational Needs in Social Communication of students with ASD? 1. Very Unsuitable 2. Unsuitable 3. Suitable 4. Very Suitable
*How would you assess the training you have received in your university career on each of the following evidence-based practices to respond to the Special Educational Needs in Social Communication of students with ASD? 1. The strategy was never mentioned and never taught 2. The strategy was mentioned incidentally 3. The strategy was mentioned and discussed 4. The strategy was mentioned and taught through direct instruction
Differential reinforcement Discrete trial training Modeling Naturalistic intervention Peer mediated interventions Prompting Reinforcement Scripting Social narratives Task analysis Time delay Videomodeling
*How would you assess the training you have received in your in-service teacher training (CPR etc.) on each of the following evidence-based practices to respond to the Special Educational Needs in Social Communication of students with ASD? 1. The strategy was never mentioned and never taught 2. The strategy was mentioned incidentally 3. The strategy was mentioned and discussed 4. The strategy was mentioned and taught through direct instruction
Differential reinforcement Discrete trial training Modeling Naturalistic intervention Peer mediated interventions Prompting Reinforcement Scripting Social narratives Task analysis Time delay Videomodeling

Example of Definition

Naturalistic Intervention

This is a collection of practices in which the teachers detect the interest of the students and provoke learning situations through the modification of the environment, the activities, or the habitual routines so that the student engages in the objective behavior and obtains the reinforcing natural consequences that it causes [12].

## Appendix B

Datos Sociodemográficos

*¿Cuál es su género? Masculino Femenino
*¿Cuál es su edad actual? Hasta 25 años (25 años o menos) Entre 26 y 35 años (35 años incluidos) Entre 36 y 50 años (50 años incluidos) Más de 50 años (de 51 años en adelante)
*¿Cuál es el grado académico más alto que usted ha alcanzado? Diplomatura Universitaria Licenciatura Universitaria Grado Máster Doctorado Otro
*¿De qué tipo es la titularidad del centro en el que trabaja y su situación laboral? Pública: funcionariado de carrera Pública: funcionariado en prácticas Pública: funcionariado interino Concertada: contratado/a Privada: contratado/a
*¿Cuál es la especialidad docente en la que se encuentra en activo actualmente? Audición y Lenguaje Pedagogía Terapéutica Otras especialidades
*Incluyendo este curso ¿Cuál es su experiencia laboral en el ámbito docente? Menos de 1 año Entre 1 y hasta 5 años (ambos incluidos) Entre 6 y hasta 10 años (ambos incluidos) Entre 11 y hasta 20 años (ambos incluidos) 21 años o más
*Incluyendo este curso ¿Cuántos años ha trabajado con alumnado con TEA a lo largo de su vida profesional? Menos de 1 año Entre 1 y 5 años (ambos incluidos) Entre 6 y 10 años (ambos incluidos) Entre 11 y 20 años (ambos incluidos) 21 años o más
*Incluyendo este curso ¿Con cuántos niños y niñas con TEA ha trabajado a lo largo de su vida profesional? Ninguno Entre 1-5 Entre 6 y 10 Entre 11 y 20 21 o más
*¿Ha recibido alguna formación específica sobre el TEA? Sí No
*¿Dónde ha recibido esa formación específica sobre el TEA (varias respuestas)? En mi carrera universitaria En cursos o talleres de extensión universitaria En cursos o talleres de formación del profesorado En cursos o talleres impartidos por otras organizaciones Mediante autoformación: libros, artículos etc. Otros

Datos sobre Práctica Basada en la Evidencia

*¿Cómo valoraría globalmente la formación que ha recibido en su etapa universitaria sobre práctica basada en la evidencia para dar respuesta a las Necesidades Educativas Especiales en Comunicación Social del alumnado con TEA? 1. Muy inadecuada 2. Inadecuada 3. Adecuada 4. Muy adecuada
¿Cómo valoraría globalmente la formación que ha recibido dentro de los programas de formación del profesorado (CPR etc.) sobre práctica basada en la evidencia para dar respuesta a las Necesidades Educativas Especiales en Comunicación Social del alumnado con TEA? 1. Muy inadecuada 2. Inadecuada 3. Adecuada 4. Muy adecuada
¿Cómo valoraría la formación que ha recibido en su carrera universitaria sobre cada una de las siguientes prácticas basadas en la evidencia para dar respuesta a las Necesidades Educativas Especiales en Comunicación Social del alumnado con TEA? 1. Esta práctica nunca fue ni mencionada ni enseñada 2. Esta práctica fue mencionada puntualmente 3. Esta práctica fue mencionada y discutida en profundidad 4. Esta práctica fue mencionada y enseñada mediante práctica directa
Refuerzo diferencial Enseñanza mediante Ensayos Discretos Modelado Intervención naturalista Intervención mediada por pares Ayuda o Instigación Reforzamiento Guiones Narrativas sociales Análisis de Tareas Retraso temporal Video modelado
¿Cómo valoraría la formación que ha recibido en su formación continua del profesorado (CPR etc.) sobre cada una de las siguientes prácticas basadas en la evidencia para dar respuesta a las Necesidades Educativas Especiales en Comunicación Social del alumnado con TEA? 1. Esta práctica nunca fue ni mencionada ni enseñada 2. Esta práctica fue mencionada puntualmente 3. Esta práctica fue mencionada y discutida en profundidad 4. Esta práctica fue mencionada y enseñada mediante práctica directa
Refuerzo diferencial Enseñanza mediante Ensayos Discretos Modelado Intervención naturalista Intervención mediada por pares Ayuda o Instigación Reforzamiento Guiones Narrativas sociales Análisis de Tareas Retraso temporal Video modelado

Ejemplo de definición

Intervención Naturalista

Se trata una colección de prácticas en las que el profesorado detectan el interés del alumnado y provocan situaciones de aprendizaje a través de la modificación del entorno, las actividades o las rutinas habituales para que el alumno se involucre en el comportamiento objetivo y obtenga las consecuencias naturales reforzantes que éste provoca [12].
